# MicroRNA-10b inhibition reduces E2F1-mediated transcription and miR-15/16 activity in glioblastoma

**DOI:** 10.18632/oncotarget.3009

**Published:** 2015-02-06

**Authors:** Nadiya M. Teplyuk, Erik J. Uhlmann, Andus Hon-Kit Wong, Priya Karmali, Meenakshi Basu, Galina Gabriely, Anant Jain, Yang Wang, E. Antonio Chiocca, Robert Stephens, Eric Marcusson, Ming Yi, Anna M. Krichevsky

**Affiliations:** ^1^ Department of Neurology, Brigham and Women's Hospital, Harvard Medical School, Boston, MA; ^2^ Regulus Therapeutics, Inc., San Diego, CA; ^3^ Department of Neurosurgery, Brigham and Women's Hospital, Harvard Medical School, Boston, MA; ^4^ Cancer Research and Technology Program, Frederick National Laboratory for Cancer Research, Leidos Biomedical Research, Inc., Frederick, MD

**Keywords:** miR-10b, E2F1 transcription, p21, glioblastoma, cell cycle

## Abstract

MicroRNA-10b (miR-10b) is commonly elevated in glioblastoma (GBM), while not expressed in normal brain tissues. Targeted inhibition of miR-10b has pleiotropic effects on GBM derived cell lines, it reduces GBM growth in animal models, but does not affect normal neurons and astrocytes. This data raises the possibility of developing miR-10b-targeting GBM therapy. However, the mechanisms contributing to miR-10b-mediated glioma cell survival and proliferation are unexplored. We found that inhibition of miR-10b has distinct effects on specific glioma cell lines. In cells expressing high levels of tumor suppressor p21WAF1/Cip1, it represses E2F1-mediated transcription, leading to down-regulation of multiple E2F1 target genes encoding for S-phase specific proteins, epigenetic modulators, and miRNAs (e.g. miR-15/16), and thereby stalling progression through the S-phase of cell cycle. Subsequently, miR-15/16 activities are reduced and many of their direct targets are de-repressed, including ubiquitin ligase FBXW7 that destabilizes Cyclin E. Conversely, GBM cells expressing low p21 level, or after p21 knock-down, exhibit weaker or no E2F1 response to miR-10b inhibition. Comparative analysis of The Cancer Genome Atlas revealed a strong correlation between miR-10b and multiple E2F target genes in GBM and low-grade glioma. Taken together, these findings indicate that miR-10b regulates E2F1-mediated transcription in GBM, in a p21-dependent fashion.

## INTRODUCTION

MicroRNA-10b (miR-10b) is the most strongly up-regulated microRNA in glioblastoma (GBM) [1–4], and is a potential therapeutic target. It is overexpressed in 90% of GBM tumors across all four subtypes, while it is undetectable in normal brain tissues. Inhibition of miR-10b is deleterious for glioma cells [2]. Importantly, both modified antisense oligonucleotides (ASO) and viral “sponge” inhibitors of miR-10b cause cell death of glioma cells but not of normal brain cells [2, 5, 6], and this may present a basis for targeted therapies.

MiRNA-10b has been proposed to promote cell cycle progression, both S-phase and mitotic transitions, as well as migration, invasion and survival of glioma cells [1, 2, 6]. MiR-10b inhibition suppresses tumor growth of subcutaneous and intracranial GBM xenografts [2, 5, 6]. CDKN1A/p21, the key tumor suppressor that restricts proliferation and induces apoptosis of transformed cells has been identified as a direct target of miR-10b in GBM, along with others that include CDKN2A/p16, BIM, TFAP2C, and TP53 [2, 5]. Due to GBM heterogeneity, however, most validated targets of the miR-10b are expressed or functional only in a subset of the tumors [7], and the detailed mechanism of miR-10b regulation of cell death and proliferation remains to be investigated.

Here we demonstrate that treatment of GBM cells with miR-10b sequence-specific inhibitors delays their progression through S-phase by down-regulation of E2F1 mediated transcription. Numerous regulated transcripts include those encoding replication factors and regulators of cell cycle, as well as miRNAs of the miR-15/16 family and their downstream post-transcriptional targets. This regulation is p21-dependent: it is observed only in GBM cells expressing high levels of the p21 protein, and can be reversed by p21 knock-down. Furthermore, analysis of GBM tumors using The Cancer Genome Atlas (TCGA) indicates a strong correlation between the levels of miR-10b and numerous E2F1 target genes. These results suggest that miR-10b is a key regulator of the E2F1 transcriptional machinery controlling cell cycle progression in GBM, and is a potential therapeutic target.

## RESULTS

### Differential cell cycle response to miRNA-10b inhibition in GBM cells

**Figure 1:**
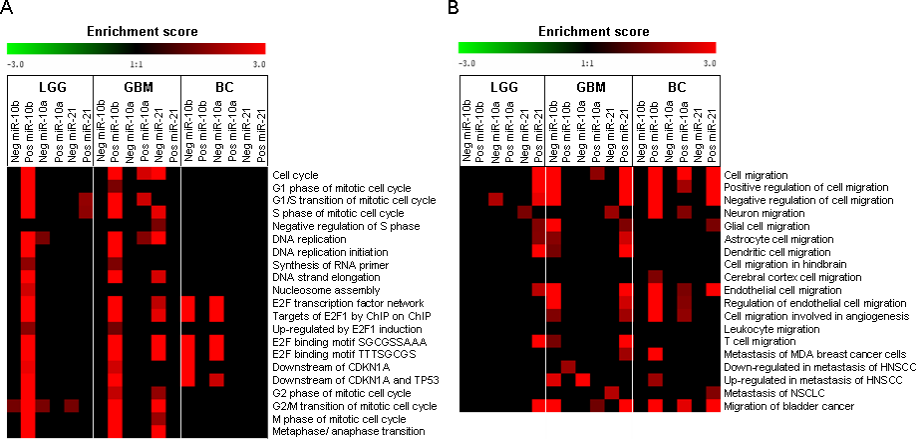
TCGA analysis: correlations between miR-10b and mRNA bioterms in GBM, LGG, and breast cancer **(A)** Pathway-level enrichment of the cell cycle and E2F1 related transcripts among the mRNAs that correlate with miR-10b in gliomas, but not in breast cancer. Significantly correlated genes (*p* < 0.0001) were assessed for enrichment of specific pathways and bioterms using multiple resources (KEGG, Biocarta, GO Biological Processes, and MSigDB). The enrichment scores are presented in the form of heatmaps, with the gradient of red color showing the level of enrichment and black corresponds to no enrichment. Enrichment for positively (Pos) and inversely correlated (Neg) genes is shown for one of the miR-10b probes used in TCGA arrays for GBM data (the other probe showed identical results). Correlations for miR-10a and miR-21, and for other cancer types, Low Grade Glioma (LGG) and Breast Cancer (BC) are shown for comparison. **(B)** Pathway-level heatmap for migration and metastasis-related bioterms was generated as in (A).

Previous work has implicated miR-10b in regulation of the GBM cell cycle. To better understand the effect of miR-10b on the cell cycle, we studied TCGA miRNA and mRNA datasets (as in [2] and analyzed the correlations between the miR-10b levels and specific cell cycle related bioterms in GBM and low-grade gliomas (LGG). Strong positive correlations have been observed between the expression of miR-10b and numerous S-phase genes, and particularly, mRNA targets of E2F transcription factors (Figure 1A, Table 1, and Supplementary Figure 1). E2F transcription plays a major role during the transition through the G1/S phase of cell cycle. Among the E2F target genes exhibiting significant correlations with miR-10b levels (* p*-value from 10^−6^ to 10^−16^) were subunits of the DNA replication complex (DNA polymerase, PCNA, DNA helicases, and ligases), regulators of nucleotide synthesis, cell cycle regulators (e.g. cyclins A2, B1, E2 and CDKs), components of chromatin assembly, PRC2 polycomb complex (EZH2, SUZ12, EED), and others. In addition, several miRNAs previously characterized as E2F transcriptional targets, also exhibited significant positive correlations with the levels of miR-10b (Table 1). This association raised the possibility of miR-10b – mediated regulation of E2F transcription. Strong correlations have been also detected between miR-10b and the genes down-regulated by p21/CDKN1A, a validated miR-10b target, suggesting that E2F- p21- targeted pathways may overlap downstream of miR-10b. In contrast, the breast cancer TCGA datasets identified no positive correlation between the miR-10b levels and cell cycle bioterms or E2F transcriptional targets (Figure 1B). Rather, in agreement with the established function of miR-10b in breast cancer in regulating invasion and metastasis [8], the expression levels of miR-10b showed correlation with cell motility, migration, and invasion, supporting a tissue-specific mechanism of miR-10b – mediated regulation [7].

**Table 1:** miR-10b positively correlates with known E2F target genes expression in glioblastoma tumors based on The Cancer Genomic Atlas (TCGA)

	r	*p* val.		r	*p* val.		r	*p* val.		r	*p* val.
Nucleotide synthesis			Replication complex			Cell cycle regulators			Mitosis		
DHFR	0.47	10^−16^	PCNA	0.39	10^−11^	FBXO5	0.45	10^−14^	PLK1	0.36	10^−9^
TYMS	0.38	10^−10^	RFC4	0.39	10^−11^	MYBL2	0.42	10^−13^	NCAPH	0.36	10^−10^
RRM1	0.35	10^−9^	LSH	0.36	10^−9^	CCNA2	0.40	10^−11^	**Other**		
DUT	0.34	10^−9^	LIG1	0.36	10^−8^	CDC25A	0.40	10^−12^	EZH2	0.48	10^−17^
RRM2	0.33	10^−8^	RMI1	0.35	10^−9^	MYCN	0.39	10^−9^	MKI67	0.34	10^−9^
TK1	0.33	10^−8^	PRIM1	0.35	10^−9^	CDK2	0.37	10^−3^	SUZ12	0.32	10^−8^
**BRCA complex**			POLE2	0.29	10^−9^	CDC2	0.37	10^−3^	SFRS2	0.31	10^−7^
BARD1	0.29	10^−8^	POLD3	0.33	10^−8^	MELK	0.36	10^−9^	EED	0.21	10^−4^
HMMR	0.27	10^−6^	RFC3	0.33	10^−8^	ATAD2	0.35	10^−8^	**miRNA E2F1 targets**:		
**Replication origin**			POLA1	0.32	10^−8^	CDC25C	0.33	10^−8^	hsa-miR-20b	0.65	10^−17^
MCM7	0.47	10^−16^	RFC2	0.29	10^−6^	CCNB1	0.32	10^−7^	hsa-miR-106a	0.63	10^−17^
TOP2A	0.46	10^−15^	FEN1	0.29	10^−6^	CDC6	0.30	10^−7^	hsa-miR-17	0.62	10^−17^
MCM2	0.41	10^−12^	BLM	0.23	10^−5^	E2F8	0.28	10^−6^	hsa-miR-20a	0.61	10^−17^
MCM4	0.38	10^−10^	**Chromatin assembly**			BTG3	0.27	10^−6^	hsa-miR-93	0.60	10^−17^
MCM10	0.35	10^−9^	HMGB2	0.43	10^−14^	SKP2	0.27	10^−5^	hsa-miR-25	0.53	10^−13^
MCM3	0.34	10^−9^	DEK	0.35	10^−9^	CCNE2	0.22	10^−4^	hsa-miR-106b	0.53	10^−12^
MCM5	0.34	10^−8^	H2AFZ	0.31	10^−7^	E2F3	0.22	10^−4^	hsa-miR-19b	0.49	10^−10^
MCM6	0.33	10^−8^	ASF1B	0.28	10^−6^	E2F1	0.20	10^−4^	hsa-miR-15b	0.49	10^−10^
ORC1L	0.31	10^−7^	CHAF1A	0.28	10^−4^	CCND2	0.20	10^−4^	hsa-miR-16	0.48	10^−10^
**Lamina assembly**			**Repair/checkpoint**								
TMPO	0.36	10^−^9	FANCL	0.32	10^−^8						
			FANCA	0.28	10^−^6						

**Figure 2:**
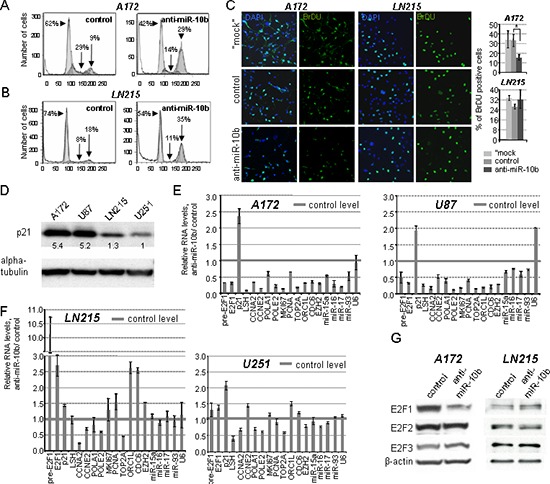
Two distinct types of cell cycle responses to miR-10b inhibition in GBM cell lines **(A–C)** miR-10b inhibitor reduces the number of cells in S-phase of the cell cycle in A172 glioma cells with high level of p21 protein, but not in LN215 cells with low p21. The percentage of cells in each phase of cell cycle was determined three days after transfections with either miR-10b inhibitor or control oligonucleotide by propidium iodide staining and flow cytometry (A, B), and incorporation of BrDU (C) **(D)** U87 and A172 GBM cell lines express high levels of p21 protein, while LN215 and U251 express lower p21, as indicated by Western blot analysis. **(E–F)** Expression of S-phase-specific E2F1 target genes is reduced 24 h after transfection with miR-10b inhibitor in A172 and U87 cells (E), but not in LN215 and U251 cells (F) Relative RNA expression levels by qRT-PCR were normalized to corresponding expression of the GAPDH mRNA. In panel E, the differences from the control values reached statistical significance (*p* < 0.05) for all genes except U6, which was used as a control. **(G)** Western blot analysis showing that expression of E2F1, but not of E2F2 and E3F3 proteins, is down-regulated in A172 cells 24 h after transfection with miR-10b inhibitor.

To better understand the effect of miR-10b on cell cycle progression of glioma cells, miR-10b activity was selectively inhibited with 2′-O-methoxyethyl (2′-O-MOE) antisense oligonucleotides in various GBM cell lines. The efficiency of this approach has been described previously [2]. Inhibition of miR-10b resulted in two distinct cell cycle responses in glioma cells. A172 and U87 cell lines exhibited reduction of cells in S-phase of the cell cycle, and accumulation of cells in G2/M, while LN215 and U251 cells showed no down-regulation of S-phase, with arrest in the tetraploid state (Figure 2A, 2B). Furthermore, a two-fold decrease in bromodeoxyuridine (BrDU) incorporation was observed in A172 cells, but not in LN215 cells (Figure 2C). In order to determine the cause of the differential response of the cell lines to miR-10b inhibition, we tested these lines for expression of the tumor suppressor p21 (CDKN1A), the potent regulator of G1/S transition [9], and one of the key direct miR-10b targets [2]. We determined that glioma cells expressing high p21 level (e.g. A172 and U87) exhibited strong S-phase response to miR-10b inhibition, whereas cells with low p21 (e.g. LN215 and U251) have not (Figure 2D). Therefore, p21 may be an important mediator of the effect of miR-10b on S-phase progression.

### MiR-10b inhibitor down-regulates E2F1-mediated transcription in GBM cells with high p21 level

**Figure 3:**
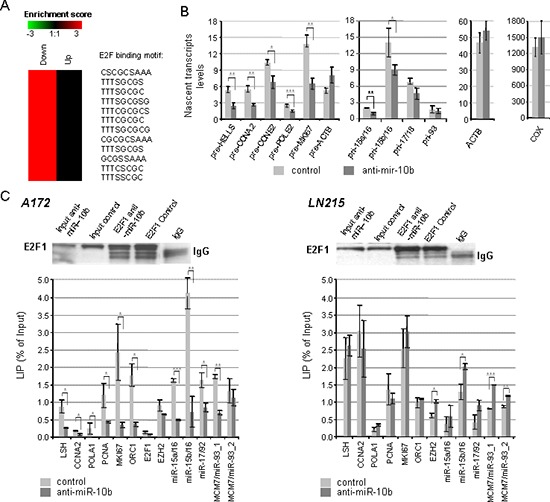
Down-regulation of E2F1 transcription by miR-10b inhibitor **(A)** Genes containing E2F1 binding sites in their promoters are significantly enriched (*p* < 0.01) among the genes downregulated at least 1.5-fold in A172 cells, 24 hours upon miR-10b inhibition, as assessed by the whole genome mRNA expression profile. **(B)** Inhibition of miR-10b represses transcription of E2F1 target genes, as shown by the ethynyl uridine incorporation assay. Relative pre-mRNA levels were quantified in the fraction of nascent transcripts by qRT-PCR, and normalized to pre-GAPDH transcript. **(C)** Binding of E2F1 to the promoters of its target genes decreases in A172, but not in LN215 cells upon miR-10b inhibition, as determined by CHIP, followed by the qRT-PCR analysis. The upper panels indicate the efficiency of E2F1 immunoprecipitation by Western blot. The lower panels represent the locus immunoprecipitation percentage. Statistical significance of the difference was determined by Student's *t* test, with *p*-values < 0.05 indicated by asterisks, *p* < 0.01 by two asterisks, and *p* < 0.001 by three asterisks.

To investigate whether the effect of miR-10b inhibition on S-phase progression is mediated by E2F transcription factors, we first studied the expression of major E2F family members in GBM cells. In A172 and U87 cells, miR-10b inhibition strongly reduced the amount of E2F1 mRNA and protein, but not that of E2F2 or E2F3, whereas none of them was reduced in LN215 or U251 cells (Figure 2E, 2F, 2G). Furthermore, multiple key E2F transcriptional targets involved in S-phase progression, as well as E2F-regulated miRNAs miR-15a/16, miR-17, and miR-93, have been down-regulated in A172 and U87 cells (Figure 2E), while few were affected in LN215 or U251 cells (Figure 2F). Most significantly, mRNA microarray analysis of A172 cells transfected with miR-10b inhibitor exhibited enrichment of E2F1 transcripts among the down-regulated genes (Figure 3A). Investigation of additional glioma cell lines and GBM-derived neurospheres provided further evidence of variable target genes' response to miR-10b depletion, which correlated with the p21 levels. More prominent down-regulation of S-phase genes occurred in LN229 and LN382 cells (expressing moderate p21 level), than in GBM4, GBM8 and BT74 cells with barely detectable p21 (Supplementary Figure 2A, 2B).

Pulse-labeling with ethinyl-uridine indicated that transcriptional rates of S-phase specific E2F targets were reduced at 18 hours after transfection with the miR-10b inhibitor (Figure 3B). Consistent with this finding, binding of E2F1 to the promoters of its target cell cycle and miRNA genes decreased dramatically upon miR-10b inhibition in A172 cells, but not in LN215 cells, as determined by chromatin immuno-precipitation (CHIP) (Figure 3C). Among others, inhibition of miR-10b reduced E2F1 binding to its own promoter, supporting the auto-regulatory mechanism of E2F1 expression. However, E2F1 binding to the promoters of several pro-apoptotic E2F1 target genes has not changed after miR-10b inhibition in A172 cells (Supplementary Figure 3). Such difference might be due to the distinct epigenetic status of target genes, and indicate that the observed effect is restricted to the regulation of cell cycle machinery. Therefore, miR-10b inhibition repressed E2F1 expression, its activity, and E2F1-driven transcription of cell cycle-related mRNAs and miRNAs in GBM cells with high levels of p21 expression, blocking progression of these cells through the S-phase of cell cycle.

### Repression of E2F1 and E2F1 target genes by miR-10b inhibition is mediated by p21

**Figure 4:**
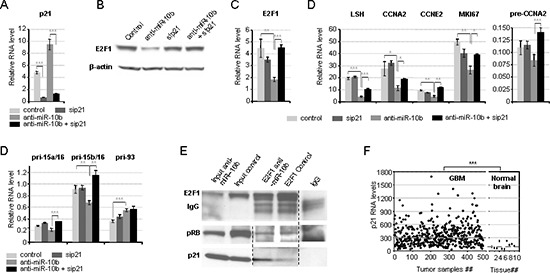
Repression of E2F1 and E2F1 target genes by inhibition of miR-10b is p21-dependent A172 cells were transfected with siRNA to p21 or control siRNA, followed by the transfection with miR-10b inhibitor or control 24 hours later. RNA and protein levels were quantified by qRT-PCR and Western blot analysis, respectively, at 48 hours. The data represent the average of biological duplicates, and control levels represent the average of three different non-specific siRNA controls. **(A)** The efficiency of siRNA-mediated knock-down of p21. **(B)** p21 knock-down causes the complete rescue of anti-miR-10b-mediated repression of E2F1 protein. **(C)** p21 knock-down causes the complete rescue of anti-miR-10b-mediated repression of E2F1 mRNA. **(D)** p21 knock-down causes complete or partial rescue of anti-miR-10b-mediated repression of E2F1 target genes. **(E)** The interaction of E2F1 with p21, but not pRB is stimulated by the miR-10b inhibition. Interaction of endogenous E2F1 with p21 and pRB repressors was analyzed by co-immuno-precipitation in A172 cells 24 hours after transfection with either miR-10b inhibitor or control. **(F)** High variability of p21 expression in GBM tumors. P21 mRNA expression levels were analyzed in TCGA GBM data. Values are shown on a linear scale. Statistical significance of the difference was determined by Student's *t* test, with *p*-values < 0.05 indicated by asterisks, *p* < 0.01 by two asterisks, and *p* < 0.001 by three asterisks.

To determine the role of p21 in the control of E2F1-mediated transcription by miR-10b, p21 expression was knocked down with small interfering RNA (siRNA) prior to miR-10b inhibition. P21 knock-down led to the rescue of E2F1 RNA and protein levels, as well as partial or near-complete rescue of many E2F1 target genes in A172 and U87 cells (Figure 4A–4D and Supplementary Figure 4A–4C). Regulation of some E2F1 target genes was completely rescued by p21 knock-down at the level of pre-mRNA, but not of mature mRNA, suggesting an additional, post-transcriptional mechanism of regulation. Consistently, p21 knock-down rescued the effect of miR-10b inhibition on S-phase progression, but not on the progression of cells through G2 phase and mitosis (Supplementary Figure 5), indicating that miR-10b regulation of G2/M is p21-independent.

The two commonly accepted effects of p21 on E2F1 transcriptional activity are inhibition of Retinoblastoma Protein (Rb) phosphorylation [9] and inhibition of E2F1 by direct binding [10]. To test these possibilities, and validate direct interaction between p21 and E2F1, we immuno-precipitated E2F1 complexes from cells transfected with anti-miR-10b or control and determined the amount of Rb1 and p21 in the E2F1 co-immunoprecipitate. The levels of p21, but not Rb1, coimmunoprecipitated with E2F1 increased upon miR-10b inhibition, likely as the result of elevated p21 expression (Figure 4E). Interestingly, total levels of Rb1 dropped upon miR-10b inhibition, suggesting a feedback mechanism developed by cancer cells to maintain the levels of E2F activity. Therefore, miR-10b appears to regulate transcription via increased p21-E2F1 binding, rather than through Rb1.

Of note, although the proposed p21-dependent control of E2F1 activity by miR-10b was observed only in a subset of cultured glioma cell lines, specifically those expressing high levels of p21, this mechanism may be widely applicable, as the majority of GBM tumor samples express high levels of p21 (Figure 4F, [11–13]). Furthermore, there is a highly significant correlation between levels of miR-10b and E2F1 transcripts across the TCGA GBM dataset (Figure 1 and Table 1), regardless of p21 expression.

### miR-10b regulates the levels and activities of the miR-15/16 cluster

**Figure 5:**
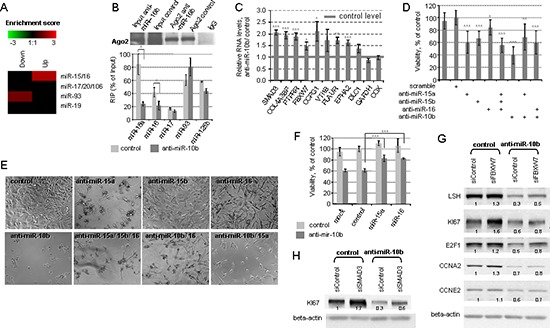
miR-10b inhibition affects cell cycle through de-repression of miR-15/16 targets **(A)** Predicted miR-15/16 family targets, but not miR-17/93 family targets are enriched among the genes de-repressed in A172 cells upon inhibition of miR-10b, as it is evident from the array whole genome expression profiling. **(B)** The amounts of Argonaut-associated miR-15a and miR-16, but not miR-17 and miR-93 decrease 24h after miR-10b inhibition in A172 cells, as detected by Ago2-CLIP. **(C)** miR-15/16 target genes are de-repressed by miR-10b inhibition in A172 cells, as detected by the qRT-PCR analysis. GAPDH and COX mRNA, not targeted by miR-15/16, are shown as controls. **(D)** Reduced cell viability 24 hours after inhibition of miR-15/16 family miRNAs in A172 cells. **(E)** Phase-contrast images were taken 48 hours after transfections with indicated miRNA inhibitors. **(F)** Protective effects of miR-15a and miR-16 over-expression on cell viability. A172 cells were co-transfected with miR-10b inhibitor and miR-15a/miR-16 precursors. Cell viability was measures 36 hours after transfection. **(G)** siRNA – mediated knock-down of FBXW7 partially rescues the levels of cell cycle proteins in miR-10b depleted cells. A172 cells were sequentially transfected with siRNAs, and miR-10b inhibitor or non-specific oligonucleotide 24 hours later. The levels of S-phase related cell cycle proteins were determined by Western blot analysis 24 hours after the inhibitor's transfection. **(H)** The effect of SMAD3 knock-down on KI67 protein level in control and miR-10b depleted cells. The experiment was conducted as described for Figure 5G. For 5 B, C, D and F, statistical significance of the difference was determined by Student's *t* test, with *p*-values < 0.05 are indicated by asterisks, *p* < 0.01 by two asterisks, and *p* < 0.001 by three asterisks.

Among the transcripts most affected by the inhibition of miR-10b in A172 and U87 cells, we identified miR-15/16. This miRNA family is one of the most abundant in gliomas [14], and a validated transcriptional target of E2F1 [15]. MiR-10b inhibition led to 3–5-fold reduction of E2F binding to the promoters of miR-15a/16 and miR-15b/16 (Figure 3C) and reduced the levels of their nascent precursor transcripts and mature miRNAs (Figure 2E and 3B). These data indicate that miR-10b regulates miR-15/16 expression through E2F1 transcriptional regulation. Furthermore, there was a significant correlation between the expression levels of miR-10b and miR-15/16 in the TCGA GBM set (Table 1 and Supplementary Figure 1). Moreover, many of the predicted targets of miR-15/16 correlated inversely with miR-10b in the TCGA GBM dataset (Table 2, [16–22]). Consistently, miR-15/16 targets were also enriched among the genes de-repressed upon miR-10b inhibition in A172 cells, shown by whole genome expression microarray profiling (Figure 5A). This suggests that miR-10b controls the levels and activities of miR-15/16 in GBM.

**Table 2:** Functional annotation of miR-15/16 target genes de-repressed upon miR-10b inhibition in A172 cells

Gene	miR-15/16 target	Ref	Correlation with miR-10b in TCGA		Function/relation to cancer
			r	*p*-value	
SMAD3	Predicted	13	−0.20	0.0007	Regulator of cell proliferation through transduction of TGF beta and BMP signals.
COL4A3BP	Predicted	14	−0.17	0.0054	Ceramide flux regulator
PTPRR	Predicted	15	−0.32	0.0000	Silenced in some cancers, potential tumor suppressor. Down-regulation activates MAP kinases. Regulates cell cycle and differentiation.
FBXW7	Validated	16, 17	−0.19	0.0023	Suppressed in GBM. Potential cell cycle suppressor. Targets Cyclin E and other cell cycle proteins to proteasome comlpex.
CCPG1	Validated	18	−0.11	0.0650	Cell Cycle Progression 1. Regulates cell cycle through interaction with Rho family factors, including CDC42.
VTI1B	Validated	18,19	−0.30	0.0000	Vesicles transport
PLAUR	Validated	18	−0.17	0.0047	Regulates migration, invasion and angiogenesis.
EPHA2	Validated	16	−0.19	0.0020	Up-regulated in cancers, including GBM, promotes GBM invasion.
DCL1	Validated	18	−0.12	0.0496	Cell cycle regulator, belongs to Rho family

To investigate whether inhibition of miR-10b modulates the activity of miR-15/16, the miRNA bound to Argonaut 2 was analyzed by cross-linking immunoprecipitation (CLIP) in A172 cells. The amount of miR-15a and miR-16 associated with Ago2 complex decreased upon anti-miR-10b treatment (Figure 5B), indicating that miR-10b modulates the activity of miR-15/16 family. Accordingly, a number of previously validated direct miR-15/16 targets have been de-repressed by miR-10b inhibition (Figure 5C); a short functional annotation of these genes is provided in Table 2. Some of these genes are known as cell cycle or tumor suppressors and therefore might be functionally involved in the cell cycle arrest caused by miR-10b inhibition.

Although the miR-15/16 family is generally considered as a tumor suppressor, and was reported to be down-regulated in many malignancies [23, 24], its expression is moderately elevated in GBM and associated with glioma progression [3, 25, 26]. To investigate the possible role of these miRNAs in GBM, we transfected A172 cells with specific inhibitors of miR-15a, miR-15b, or miR-16, alone or in combination with the miR-10b inhibitor. MiR-15a, -15b and -16 inhibitors moderately decreased the viability of glioma cells (Figure 5D, 5E), suggesting an unusual tumor-promoting role of miR-15/16 family in GBM. There was no additive effect for miR-15/16 and miR-10b inhibitors on cell viability, implying a common pathway that includes both miR-10b and miR-15/16. Conversely, miR-15a and miR-16 mimics protected glioma cells against the anti-miR-10b and improved cellular viability (Figure 5F).

To further investigate the functional contribution of miR-15/miR-16 target genes to the miR-10b-mediated cell cycle phenotype, we focused on three genes implicated in the regulation of cell cycle: FBXW7/AGO, SMAD3, and CCPG1 (Table 2). These selected miR-15/16 targets have been knocked-down by cognate siRNAs prior to the miR-10b inhibition, and the efficiency of RNAi is presented in Supplementary Figure 6. We found that FBXW7 knock down partially rescued the miR-10b mediated down-regulation of many critical S-phase proteins, such as E2F1, LSH, Ki67, Cyclin A2, and Cyclin E2 (Figure 5G). Consistent with the function of FBXW7 as ubiquitin ligase, this rescue occurs at the protein but not mRNA expression level (Supplementary Figure 7). In addition, knock-down of SMAD3 led to the partial rescue of anti-miR-10b-mediated down-regulation of proliferative marker Ki67, but not other tested cell cycle genes (Figure 5H). We have not detected any significant effects of the CCPG1 knock-down on the expression of tested cell cycle genes (Supplementary Figure 8). These data confirm the functional contribution of miR-15 and miR-16 targets to the miR-10b/E2F1 signaling and highlight a multifactorial regulation of cell cycle progression in GBM cells, that includes miRNA, transcriptional, and protein stability regulatory layers. These results also suggest a complex relationship between principal miRNA regulators and indicate an unanticipated tumor-promoting role for the established tumor-suppressor family of miRNAs.

## DISCUSSION

MicroRNA regulate a wide range of biological processes, including development, differentiation, and phenotype maintenance. Targeting a specific set of genes to execute a developmental program, some miRNAs may act either as oncogenes or tumor suppressors, and thus represent useful targets in cancer therapy. Numerous pre-clinical, and several clinical studies have suggested the efficacy of miRNA inhibitors in disease [27], recently reviewed in [28].

It has been previously reported that miR-10b, a miRNA most robustly up-regulated in GBM, affects both cell cycle and survival of GBM cells [2]. Initially found to be an oncogene in metastatic breast cancer [8, 29], miR-10b appeared to play a growth-promoting role in the broad range of different cancers, including GBM, lung, ovarian, pancreatic, hepatic, thyroid, gastric, colorectal, and nasopharyngeal [5, 6, 30–39]. In tumor cells, miR-10b can act through pleiotropic mechanisms, including stimulation of cell proliferation, survival, migration, and invasion. Importantly, inhibition of miR-10b reduces the growth of cultured glioma cells and prolongs survival in mouse xenograft models of GBM [2, 5, 6]. The low or absent expression in the normal brain, in contrast with high level expression in GBM and low-grade glioma makes miR-10b an attractive therapeutic target. Better understanding of the mechanism of action of miR-10b is crucial in the development of new GBM treatments.

In this work we have further dissected the effects of miR-10b inhibition in glioma. We provide evidence that miR-10b regulates a major transcription factor, E2F1, driving cell cycle progression through the S-phase, and thus promoting proliferation. Alterations along the E2F-signaling axis, such as deactivation and mutations of Rb1 and p16/CDKN1A, and Cdk4/6 amplification, are frequently found in GBM [40]. Our work has identified miR-10b as additional critical regulator of the E2F signaling. The association between miR-10b and E2F1 transcription, observed in TCGA not only in GBM but also in LGG, implies that miR-10b-E2F1-regulated gene expression is important at early stages of cellular transformation toward malignant glioma. miR-10b regulation of E2F1 is largely mediated via single direct target and key tumor suppressor gene CDKN1A/p21, which directly binds to and represses E2F1. The downstream cascade involves numerous E2F1 transcriptional targets critical for the chromatin remodeling, regulation of cell cycle machinery, and DNA replication. Among them are KI67 and proliferating cell nuclear antigen (PCNA), commonly accepted proliferation markers associated with course of various cancers, including malignant gliomas [41–44]. Strongly regulated by miR-10b is DNA helicase LSH, one of the key factors defining genome-wide DNA methylation pattern, expressed in undifferentiated cells. LSH promotes proliferation and prevents senescence in multiple malignancies, while its disruption causes a premature aging phenotype [45]. An additional E2F1 target regulated by miR-10b, EZH2, is the catalytic subunit of Polycomb Repressor complex 2, overexpressed in various malignancies, including GBM, that inhibits differentiation, activates cell cycle, increases cell motility, and enhances self-renewal and tumor initiating capacity [46–49]. Therefore, by restraining E2F1 transcription, miR-10b inhibition coordinately reduces the levels of many critical tumorigenic factors in glioma, some of which represent promising candidates for targeted molecular therapies. Although miR-10b regulation of E2F1 transcription occurs only in a subset of cultured glioma cell lines, the generally high levels of p21 expression in the majority of human GBM tumors [11–13], and significant positive correlations between the levels of numerous E2F-regulated genes and miR-10b in TCGA, suggest that this mechanism is widespread in GBM. Nevertheless, additional mechanisms involved in G2/M arrest and cell death upon miR-10b inhibition, especially those common for p21-low cells, should be further investigated.

Our data also indicate that a miRNA may amplify its function by recruiting or regulating other – secondary miRNAs. We demonstrate that the miR-15/16, and to a lesser extent the miR-17/93 cluster is upregulated by miR-10b. MiR-17/93 is a family of oncogenic miRNAs, induced by E2F1, and, in turn, repressing E2F1 in a feedback loop [50, 51]. MiR-15/16 are frequently lost or reduced in malignancies, and are considered to be tumor suppressors [23, 52, 53]. In GBM, however, they are up-regulated and their expression is associated with glioma progression [3, 25]. miR-10b inhibition strongly reduces expression of miR-15a, -15b, and -16 members of the same family from two E2F1-driven promoters mapped at chromosomes 13q14.2 and 3q25.3. This leads to the significantly reduced association of miR-15/16 with Ago2 effecter complexes and de-repression of many miR-15/16 mRNA targets. Given the abundance of miR-15 and -16 in GBM, this pathway may have profound effects on the glioma transcriptome.

A consequent question is whether the activation of miR-15/16 by miR-10b serves as a negative feedback on proliferation, or miR-15/16 paradoxically promote GBM growth, in a tissue-specific manner. The suppression of glioma cell growth observed in miR-15/16 inhibition experiments, as well as the partial rescue of glioma viability provided by miR-15/16 mimics from anti-miR-10b, support the tumor-promoting role for these miRNAs in GBM, and suggest them as mediators of the miR-10b function.

Some of the de-repressed miR-15/16 targets are known as potent regulators of cell cycle (Table 2). Particularly, FBXW7 (also known as CDC4 or Archipelago) is E3 ubiquitin ligase that targets cell cycle - related proteins, such as cyclin E, for degradation. Importantly, we found that knock-down of FBXW7 increases the levels of several cell cycle proteins, such as E2F1, LSH, KI67, Cyclin A2 and Cyclin E2, both in basic conditions, as well as in miR-10b – depleted glioma cells. As expected, the regulation of protein (but not corresponding mRNA) expression was observed, supporting the role for FBXW7 as a broad regulator of cell cycle proteins' degradation. The miR-10b regulated expression of FBXW7, and FBXW7 effects on the cell cycle machinery in glioma cell are in agreement with the reports showing that FBXW7 is frequently down-regulated in GBM [54], and its overexpression blocks proliferation of glioma cells [55]. In addition, knock-down of SMAD3 increases the levels of proliferation marker KI67 in miR-10b depleted cells. SMAD3, a mediator of TGF-beta signaling, is known to interact with, and regulate APC/C ubiquitin ligase [56]. APC/C, in turn, is capable of delaying S-phase progression, by targeting Germinin, DBF4 and other critical cell cycle substrates [57].

**Figure 6:**
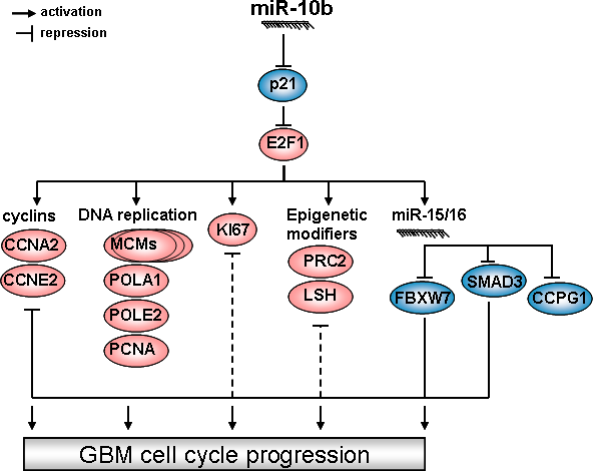
The hierarchy of miR-10b control over glioma cell cycle progression Blue ovals depict tumor suppressive factors down-regulated by miR-10b, while red indicate factors indirectly activated by miR-10b.

In conclusion, our data indicate that activation of miR-15/16 family through miR-10b/p21/E2F1 regulatory axis results in attenuation of FBXW7 and SMAD3, and therefore, increased levels of S-phase specific cell cycle proteins, providing support for cell cycle progression, in addition to the transcriptional up-regulation of these genes by E2F1 (Figure 6).

An important question is whether miR-10b also regulates cell cycle and E2F1 transcription in other extracranial cancers. MiR-10b has been studied intensively in breast carcinoma, where it is known to regulate invasion and metastasis, rather than cell proliferation [8]. Consistently, TCGA analysis showed no correlation between miR-10b and cell cycle bioterms, and rather negative correlation between miR-10b and E2F transcription in breast carcinoma (probably reflecting silencing of the S-phase genes and cell cycle exit, required for the cancer cell migration and invasion), and reiterating high tissue and context dependence of miR-10b regulation. Therefore, our data provide further support for the idea that miR-10b has very distinct functions in glioma and other cancers such as breast carcinomas [7].

The coordinated regulation of numerous E2F1 transcription targets, both mRNAs and miRNAs, by miR-10b in glioma, suggests this miRNA as a powerful regulator of glioma growth and, particularly, E2F1–driven oncogenesis. Of note, our results rely largely on the sequence-specific chemically modified miR-10b oligonucleotide inhibitors. This is a convenient and highly reproducible approach that has the potential as an experimental therapy. However, toxic effects arising from the chemical modifications and off-target effects cannot be ruled out and should be carefully controlled. Complementary methods including miRNA gene knock-out, overexpression, as well as genetic epistasis should be used in further studies.

## MATERIALS AND METHODS

### Analysis of GBM, LGG, and breast cancer *in vivo* data from TCGA

The TCGA miRNA and mRNA expression data (either RNAseq or microarray) and metadata for various cancer patients were downloaded from the following NCI TCGA data portal: https://tcga-data.nci.nih.gov/tcga/, or Firehose data download website at Broad Institute: http://gdac.broadinstitute.org/. In general, normalized and processed level-3 of miRNAseq and mRNAseq or level-2 data of microarray were downloaded, combined, and processed with customized R scripts (http://www.r-project.org). Specifically, for breast cancer (BRCA), Illumina GA or HiSeq platform mRNA RNAseq and miRNAseq level-3 data were downloaded and used for analysis; for Brain Lower Grade Glioma (LGG), Illumina HiSeq platform mRNA RNAseq and miRNAseq level 3 data were downloaded and used for analysis; for Glioblastoma multiforme (GBM), most recent TCGA microarray level-2 data for mRNA and miRNA were downloaded and used for analysis. The lists of mRNAs correlated with selected miRNAs (i.e, miR-10b, miR-10a, miR-21) were further analyzed for pathway enrichment patterns for multiple cancer types (GBM, LGG, BRCA) using in-house tool PPEP analysis pipeline [58] using KEGG (http://www.genome.jp/kegg/), Biocarta (http://www.biocarta.com/) and Gene Ontology database (GO, http://www.geneontology.org), MSigDB (http://www.broadinstitute.org/gsea/msigdb/index.jsp) as previously described [2].

### Cell culture and transfections

Human GBM cell lines were obtained from American Type Culture Collection (ATCC), cultured in DMEM supplemented with 10% FBS (Gibco), and passaged by trypsinization.

MiRNA inhibitors used in this study were developed and provided by Regulus Therapeutics, Inc., San Diego, CA. The following full-length antisense 2′-O-MOE-modified oligonucleotides with phosphate backbone were used for miRNA inhibition: mR-10b 5′-CACAAATTCGGTTCTACAGGGTA-3′, miR-15b 5′-TGTAAACCATGATGTGCTGCTA-3′, miR-16 5′-CGCCAATATTTACGTGCTGCTA-3′, miR-15a 5′-CACAAACCATTATGTGCTGCTA-3′, and the oligonucleotide 5′-ACATACTCCTTTCTCAGAGTCCA-3′ was used as the chemistry-matched control. siRNAs for p21 knock-down were described previously [2]. The cells were transfected with miRNA inhibitors (using Lipofectamine 2000, Invitrogen) or siRNAs (using Oligofectamine) at 50 nM final concentration, according to manufacturer's instructions. For rescue experiments, sequential transfections were performed, first by transfecting the siRNA, followed by the miRNA inhibitors 24 h later.

### Cell cycle analysis by propidium iodide staining and flow-cytometry

Cells were trypsinized to single cells suspension, washed with PBS, and fixed in 75% ethanol in −20°C for 24 hours. Cells were further washed with PBS-0.1% Triton X-100 and stained in 1 ml of 50 μg/ml propidium iodide (Sigma-Aldrich) and 100 μg/ml RNAse A (Sigma-Aldrich) in PBS-0.1% Triton X-100 at room temperature for 30 minutes. Aggregated cells were removed by a cell strainer (BD Falcon). Flow cytometry was performed on an LCRII (BD Biosciences), and data analyzed using FlowJo software. Typically, 20,000 cells per sample were acquired.

### S-phase quantification by bromo-2′-deoxyuridine (BrDU) incorporation

A172 and LN215 cells were plated on coverslips and transfected with 50 nM anti-miR-10b or control oligonucleotides. Cells were pulse-labeled with 10 μM BrdU 30 hours after transfection for 2 hours. Immediately after the labeling, the cells were washed, fixed, and stained for BrdU using a BrdU labeling and detection kit (Roche), according to the manufacturer's instructions. 4′,6′-diamino-2-phenylindole (DAPI) staining was used to determine the total number of cells. The images were taken with a Zeiss LSM710 confocal microscope on low magnification (10X). The average percentage of BrdU positive cells was quantified in four different microscope fields using ImageJ software.

### Western Blot Analysis

Western blot analysis was performed by standard procedure (see Supplementary Methods). The following primary antibodies were used: anti-p21, 2947 (12D1), anti-E2F1 3742, anti-Rb1, 9313, anti-Cyclin A2, 4656, anti-Cyclin E2, 4132 (Cell Signaling), anti-E2F2 sc-633 (C-20), anti-E2F3 sc-878 (C-18), anti-LSH sc-28202 (H-240) (Santa Cruz Biotechnology), anti-KI67 ab16667, anti-beta-Actin ab3280 (Abcam), anti-alpha-Tubulin (Sigma, T9026, DM1A).

### Analysis of gene expression by quantitative real time reverse transcriptase-polymerase chain reaction (qRT-PCR)

For gene expression analysis, total RNA was isolated from cells or tissues using commercial reagents (Trizol and MirVana, Invitrogen). For primary transcripts and mRNA expression analysis, 5 μg of total RNA was subjected to DNAse I digestion and purification using a commercial RNA purification kit (Zymo Research). Aliquots of RNA (1 μg) were used in 20 μl of reverse transcription (RT) reaction using a cDNA synthesis kit with random hexamers primers (SuperScript VILO, Invitrogen). The cDNA was diluted 40 times, and 4 μl was used in 20 μl of qPCR reaction using Real-Time PCR (ViiA™ 7 with SYBR Green Master Mix, Applied Biosystems), with 0.5 pmol/μl primers. Sequences of pre-mRNA, mRNA and pri-miRNA primers used are listed in the Supplementary Table 1.

### Analysis of genes transcription by 5-ethynyl uridine pulse-labeling

The cells were transfected with the miR-10b inhibitor or control oligonucleotide, and pulse-labeled 18 hours later with 0.5 mM 5-ethynyl uridine for 30 minutes. For the analysis of transcriptional activity, a nascent RNA capture kit (Click-iT, Invitrogen) was used according to the manufacturer's instructions.

### Whole genome expression profiling by microarray

A172 cells were transfected with the miR-10b inhibitor or control oligonucleotide, and collected 24 h post-transfection. Total RNA was isolated and the whole genome microarray expression profiles were conducted using a commercial array (U133, Affymetrix). Data processing and sample comparisons were performed using an open source library for statistical analysis (Bio-Conductor library for R environment). The multiarray average expression measure from the software supplied by the manufacturer of the array was applied to calculate the average value of signal. Following multiarray average background correction, array values were subjected to quantile normalization assuming identical signal distributions in each of the arrays. Statistically significant differences between probe sets were evaluated using Student's *t* test (*p* < 0.05).

### Chromatin immuno-precipitation, Ago-CLIP and co-immuno-precipitation

Immuno-precipitation was conducted for proteins, RNA and DNA interaction analysis simultaneously according to the standard procedure (see detailed description in Supplementary Methods). Briefly, the cells were transfected with miR-10b inhibitor or control oligonucleotide. 24 hours later, the cells were washed, cross-linked with 0.4% formaldehyde, and lysed in RIPA buffer by sonication.

The following antibodies (4 ug) were used for immuno-precipitation: E2F1 (Upstate, KH20 and KH95), Ago2 (Wako Biologicals, 4G8), or non-specific mouse IgG control antibodies (Upstate). Immuno-precipitation was performed with 50 μl of antibody-coated Protein G Dynabeads (Invitrogen) in 1 ml of cell lysates containing 1 mg total protein, in the presence of protease and RNAse inhibitors, at 4°C overnight. After immuno-precipitation, the beads were washed, cross-linking was reversed and DNA, RNA and proteins isolated.

The relative binding of E2F1 to the promoters was calculated as Locus Immunoprecipitation Percentage (LIP) using the following formula: LIP (%) = (2^(Ct Input − Ct pull down)^ − 2^(Ct Input − Ct IgG)^) * 100%.

Sequences of CHIP primers are listed in the Supplementary Table 2.

## SUPPLEMENTARY METHODS


