# Supplementary material for: MicroRNA-10b inhibition reduces E2F1-mediated transcription and miR-15/16 activity in glioblastoma
Source: Oncotarget. 2015 Feb 6;6(6):3770–83. doi: 10.18632/oncotarget.3009 (PMC4414152; doi:10.18632/oncotarget.3009)
Supplement: Supplementary file 1 [file oncotarget-06-3770-s001.pdf]

SUPPLEMENTARY METHODS

Western blot analysis

For Western blotting, cells were lysed and boiled in 100 µl of Direct Lysis buffer (50 mM Tris-HCl, pH 6.8, 2% SDS, 10% glycerol, 12% urea, 25 µM MG132, 100 mM dithiothreitol, bromphenol blue and Complete protease inhibitors, (Roche Applied Science). Aliquots of each lysate (20 µl) were separated on a 4–20% Tris-Glycine Gel (Novex, Invitrogen). Gels were wet-transferred to PVDF membranes (Pall). Tris-buffered saline (TBS) with 5% nonfat dry milk was used for 1 hour to block nonspecific protein binding. Membranes were incubated with primary antibodies in TBS-0.1% Tween 20 with blocking reagent at 4°C, overnight. Horseradish peroxidase-labeled antibodies were detected with a commercial assay (ECL, GE Healthcare).

The following primary antibodies were used: anti-p21, 2947 (12D1), anti-E2F1 3742, anti-RB, 9313, (Cell Signaling) at 1:1000 dilution with 5% BSA as blocking reagent, anti-E2F2 sc-633 (C-20), at 1:200 dilution, anti-E2F3 sc-878 (C-18), 1:100, beta-Actin, sc-1615-R (C-11), 1:2000, (Santa Cruz Biotechnology) with 1% nonfat dry milk as blocking reagent, alpha-Tubulin, (Sigma, T9026, DM1A) at 1:10,000 dilution with 1% nonfat dry milk were used. Commercial HRP-labeled secondary antibodies were used (Cell Signaling) at 1:1,000 dilution for 1 hour RT in PBS, 0.1% Tween 20 with 1% nonfat dry milk.

Analysis of gene transcription rates by nascent RNA capture

For the analysis of transcriptional rate, a nascent RNA capture kit (Click-iT, dInvitrogen) was used in accordance with the manufacturer's instructions. Briefly, cells were pulse-labeled with 0.5 mM 5-ethynyl uridine for 30 minutes, further washed with PBS, and total RNA was isolated using Trizol. The newly synthesized RNA fraction was labeled in the reaction of 5 µg of total RNA with 0.5 mM biotin azide for 30 minutes with gentle vortexing. After the reaction, RNA was ethanol precipitated, washed with 75% ethanol, and the biotinylated RNA fraction was separated from 400 ng of total RNA using 25 µl of washed beads (Dynabeads MyOne Streptavidin T1, Invitrogen) for 30 minutes with gentle vortexing.

Chromatin immuno-precipitation, Ago-CLIP and co-immuno-precipitation

Immuno-precipitation was conducted according to the standard procedure, simultaneously for proteins, RNA, and DNA interaction analysis. A172 and LN215 cells were transfected with miR-10b inhibitor or control oligonucleotide. 24 hours later, cells were washed with PBS twice and cross-linked with 0.4% formaldehyde for 10 minutes at room temperature. The reaction was terminated with 0.128 M glycine and the formaldehyde was washed out twice with PBS. The cells were harvested in PBS in the presence of protease inhibitors (Mini Cocktail, Roche) and centrifuged at 1000 g for 3 minutes at 4°C.

Cell pellets were re-suspended in RIPA buffer complemented with 1% NP-40, 0.5% Na deoxycholate and CPI and 100 U/ml RNase inhibitor (RNaseOUT,Invitrogen), incubated on ice for 10 minutes. Samples were sonicated four times for 20 seconds. Cell lysates were cleared by centrifugation at 8000 g for 1 minute at 4°C. Protein concentration of the lysates was measured using a commercial kit (BCA, Thermo Scientific), and aliquots of lysates containing 1 mg of total protein were taken as input samples. Lysates were frozen in liquid nitrogen and stored at −80°C.

For immuno-precipitation, Protein G beads (Dynabeads, Invitrogen) were washed and coated with 4 µg of mouse monoclonal antibody to E2F1 (Upstate, KH20 and KH95), or mouse monoclonal antibodies to Ago2 (Wako Biologicals, 4G8), or non-specific mouse IgG control antibodies (Upstate) following the manufacturer's instructions. Immuno-precipitation was performed with 50 µl of antibody-coated beads in 1 ml of cell lysates, 1 mg total protein, CPI and 100 U/ml RNase inhibitor, at 4°C overnight with rotation.

After immuno-precipitation, the beads were washed with low salt buffer, and high salt buffer twice for 10 minutes, then, high salt buffer, low salt buffer and Tris-EDTA buffer, pH 8.0. The composition of low salt wash buffer was 20 mM Tris-HCl, pH 8.0, 150 mM NaCl, 0.1% SDS, 1% Igepal, 2 mM EDTA, 0.5% Na deoxycholate, CPI and 100 U/ml RNAse inhibitor. The composition of high salt buffer additionally included 1 M NaCl.

For protein isolation, the beads were re-suspended in 25 ul of PBS with CPI, 50 µl of 2X SDS sample loading buffer was added, and proteins were separated from beads by incubating for 10 minutes at 70°C. 25 µl of protein lysates were analyzed by Western blot.

For RNA and DNA isolation, cross-linking was reversed by rotating the beads at 65°C for 5.5h in 150 µl of reversal buffer that contained 10 mM Tris-HCl, pH 7.0, 5 mM EDTA, 1% SDS, 10 mM DTT, 100 U/ul of RNAse inhibitor and 1 g/l of proteinase K (New England Biolabs).

For RNA isolation, the samples were lyophilized and total RNA was extracted using a commercial kit (miRVana, Ambion), according to the protocol. 1 µl of each total RNA sample (IP or Input) was used for

miRNA detection, while for mRNA analysis cDNA was synthesized from 14 µl of total RNA.

DNA was isolated by the standard procedure, using extraction with phenol/chloroform/isoamyl alcohol. DNA was precipitated with 0.9 volume of isopropanol, 0.1 volume of 7.5 M ammonium acetate, and 1 µg of glycogen (Invitrogen). DNA pellets were washed with 70% ethanol, air-dried and re-suspended in 20 µl of TE buffer, pH 8.0 at 65°C for 10 minutes. The relative binding of E2F1 to the promoters was calculated as Locus Immunoprecipitation Percentage (LIP) using the following formula: LIP (%) = $(2^{(\mathrm{Ct\ Input} - \mathrm{Ct\ pull\ down})} - 2^{(\mathrm{Ct\ Input} - \mathrm{Ct\ IgG})}) * 100\%$.

SUPPLEMENTARY FIGURES AND TABLES

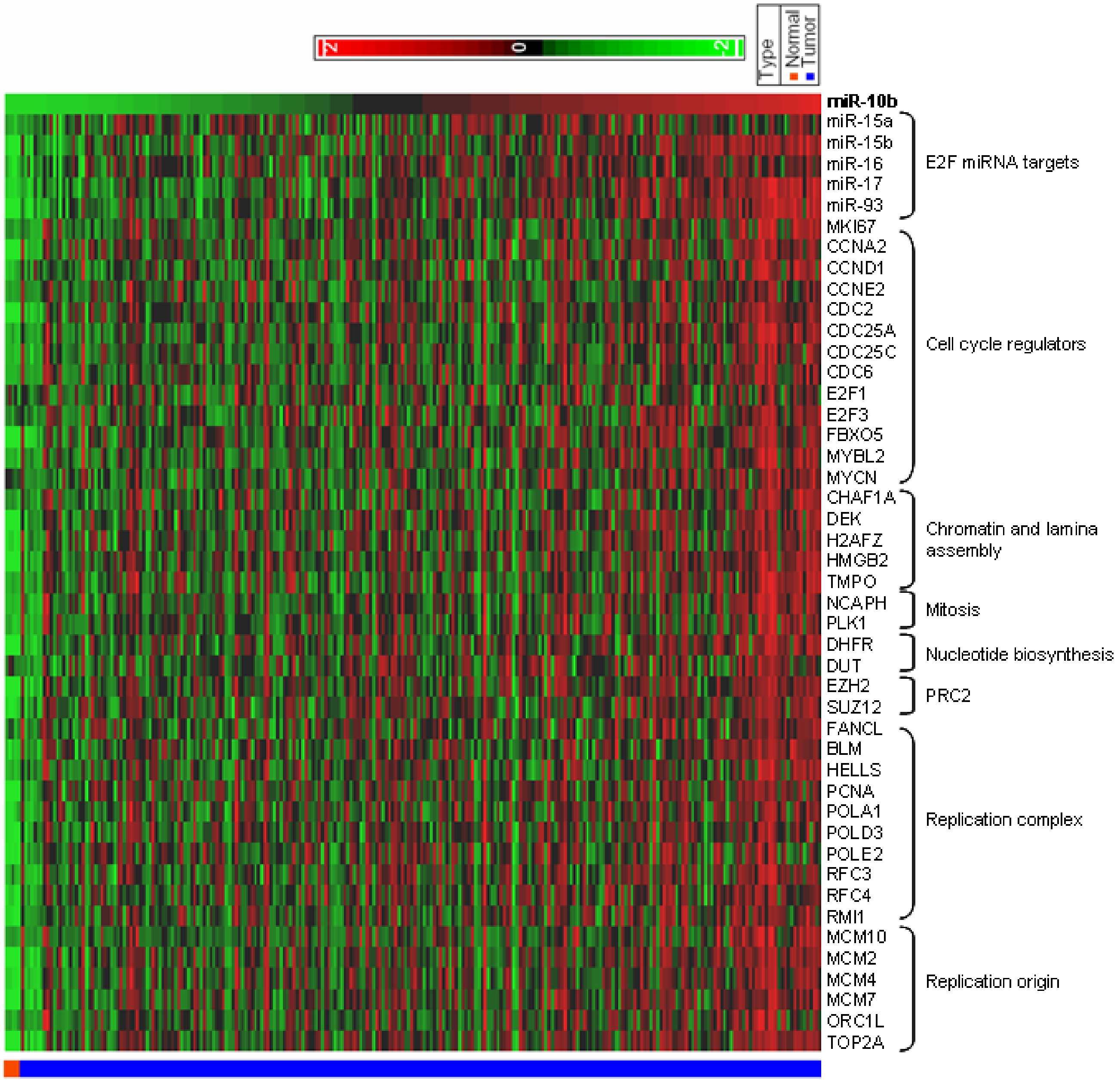

Supplementary Figure 1: The gene-level heatmap shows overall correlation between the levels of miR-10b and validated E2F target genes. Relative expression levels (z-scores) of individual E2F transcriptional mRNA and miRNA targets (in rows) are shown in the gene-level heatmap for all TCGA GBM samples, including tumors and normal brains. Human samples (in columns) are sorted by the miR-10b expression levels.

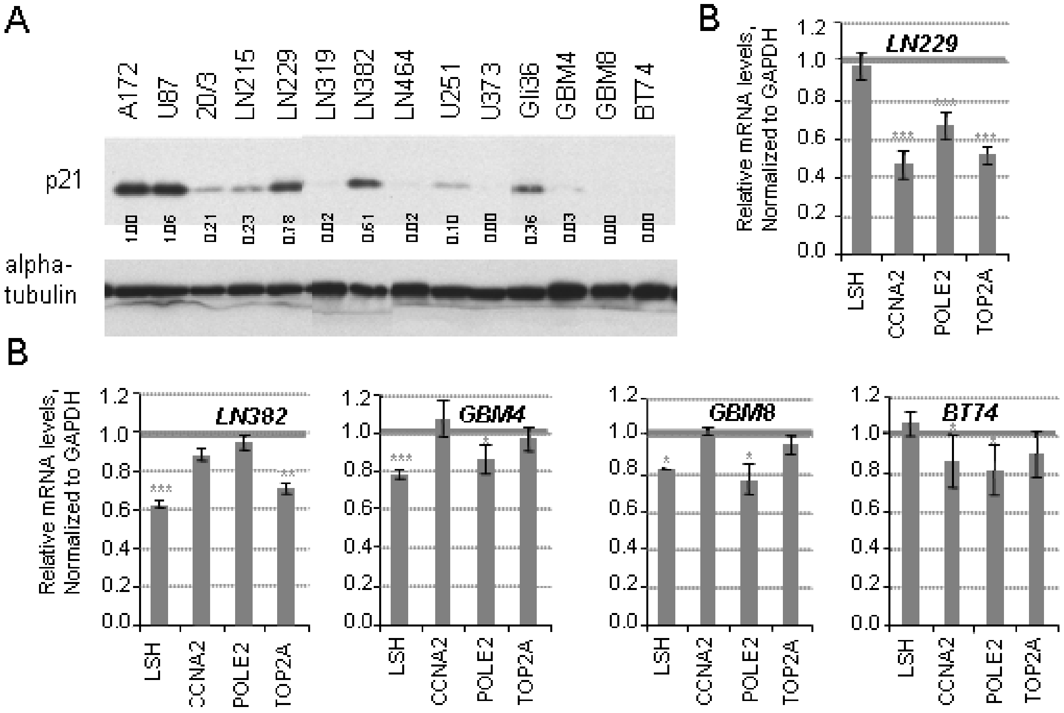

Supplementary Figure 2: Diverse p21 levels and various responses to miR-10b depletion in glioma cell lines. (A) p21 protein expression was detected in glioma cell lysates by Western blot analysis. **(B)** Regulation of cell cycle related E2F target genes by miR-10b inhibitor in different GBM cell lines was assessed by qRT-PCR.

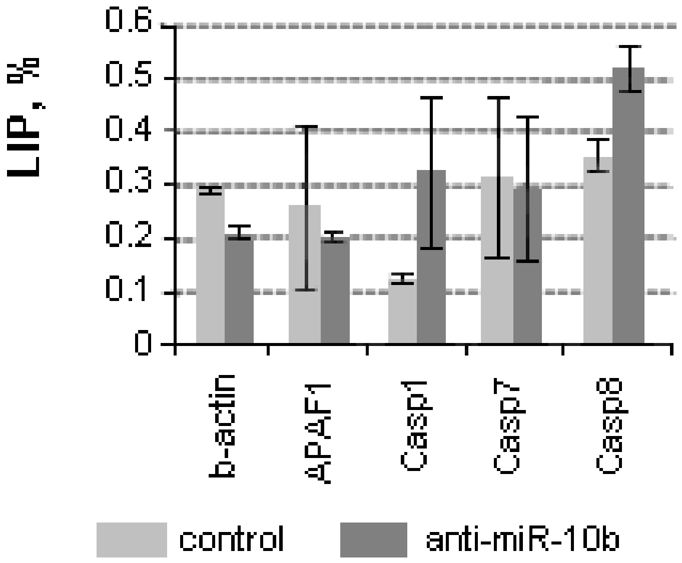

Supplementary Figure 3: E2F1 binding to the promoters of apoptosis – related E2F target genes does not decrease upon miR-10b depletion in A172 cells. The binding was examined by CHIP, followed by the qRT-PCR analysis. LIP: Locus Immunoprecipitation Percentage.

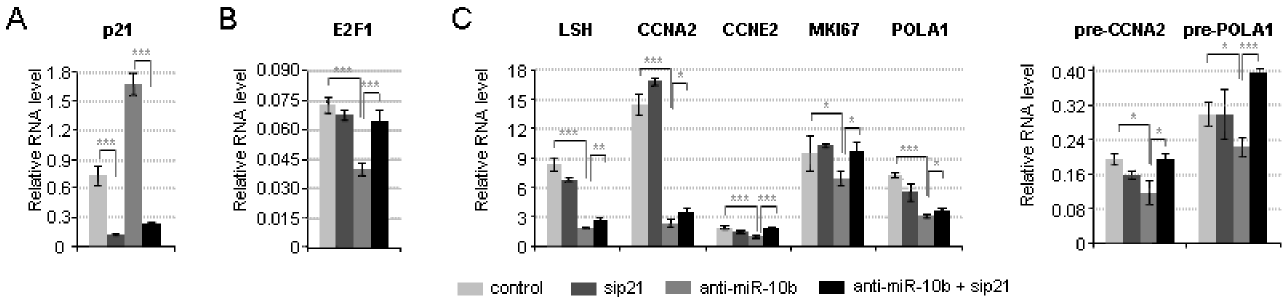

Supplementary Figure 4: Repression of E2F1 and E2F1 target genes by anti-miR-10b depends on p21 in U87 cells. U87 cells were transfected with siRNA to p21 or control siRNA, followed by the transfection with miR-10b inhibitor or control 24 hours later. RNA and protein levels were determined by qRT-PCR and Western blot analysis, respectively, at 48 hours. The data represent the average of biological duplicates, and control levels represent the average of three different non-specific siRNA controls. **(A)** The efficiency of siRNA-mediated knock-down of p21. **(B)** p21 knock-down causes the complete rescue of anti-miR-10b-mediated repression of E2F1 mRNA. **(C)** p21 knock-down causes complete or partial rescue of anti-miR-10b-mediated repression of E2F1 target genes. Values are shown on a linear scale. Statistical significance of the difference was determined by Student's t test, with *p*-values < 0.05 indicated by asterisks, *p* < 0.01 by two asterisks, and *p* < 0.001 by three asterisks.

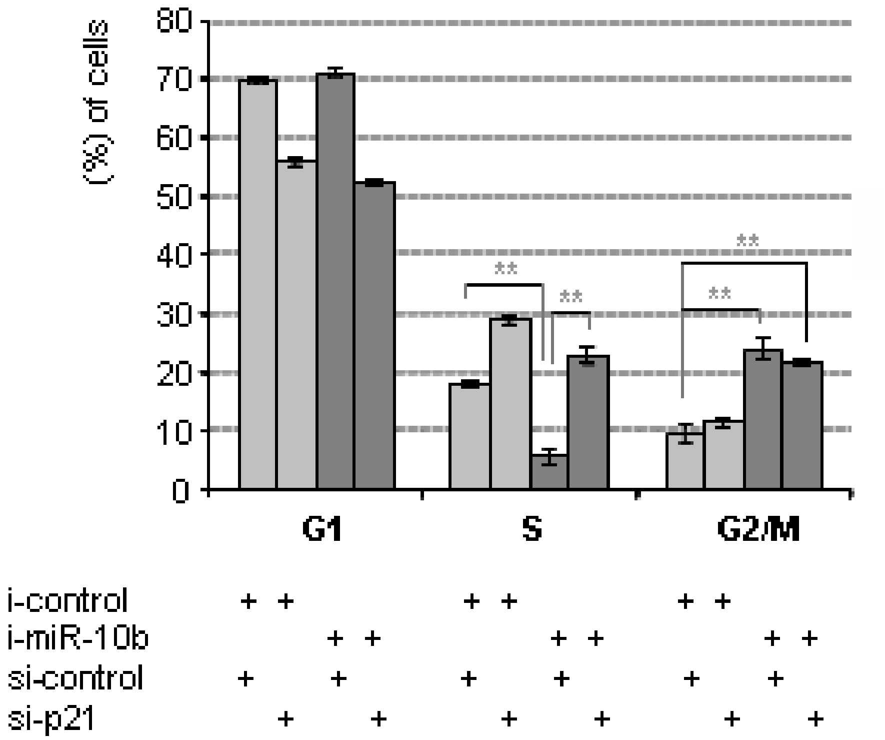

Supplementary Figure 5: Decrease in S-phase progression in miR-10b depleted cells depends on p21 level. A172 cells were transfected with siRNA to p21 or control siRNA, followed by the transfection with miR-10b inhibitor or control 24 hours later. The percentage of cells in each phase of cell cycle was determines 48 h after siRNA transfection by propidium iodide staining and FACS analysis. The data represent the average of biological duplicates. Statistical significance of the difference was determined by Student's t test, with *p*-values < 0.05 indicated by asterisks, *p* < 0.01 by two asterisks, and *p* < 0.001 by three asterisks.

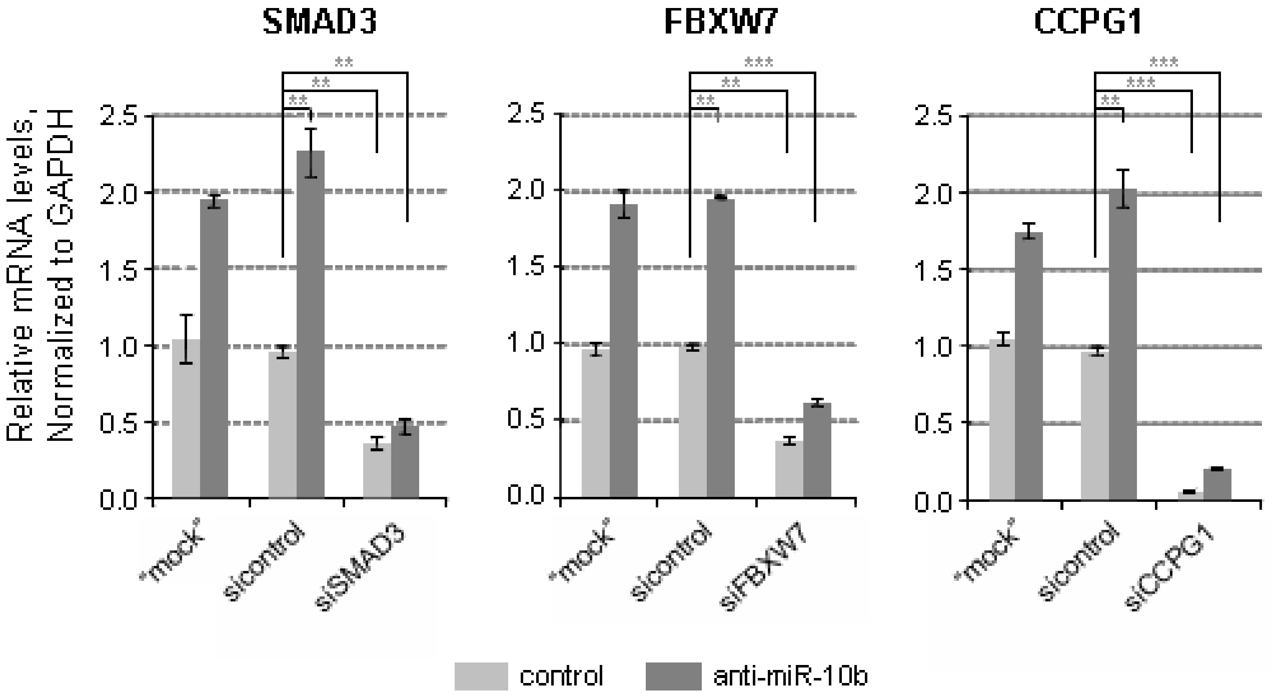

Supplementary Figure 6: The efficiency of siRNA – mediated knock-down of miR-15/16 targets. mRNA levels of SMAD3, FBXW7 and CCPG1 were quantified 48 hours after siRNA transfection to A172 cells by qRT-PCR.

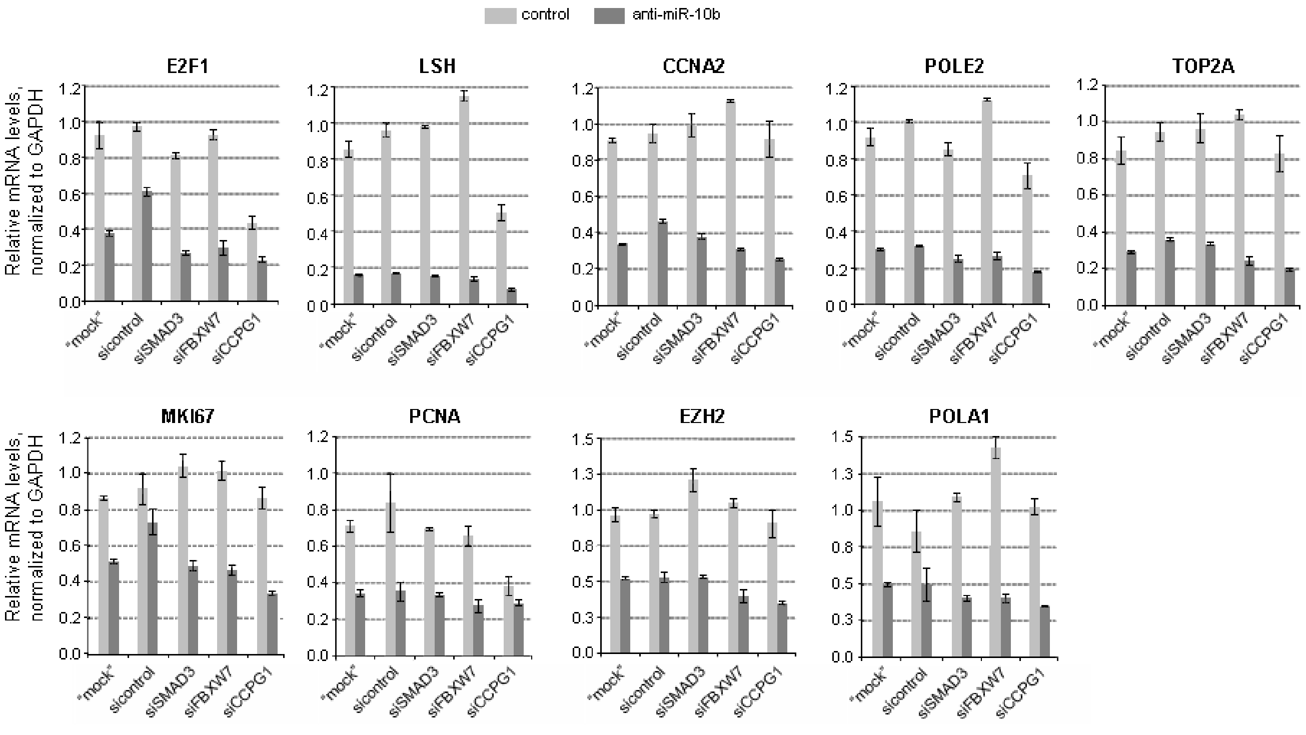

Supplementary Figure 7: The effect of miR-15/16 targets knock-down on mRNA expression in control and miR-10b – depleted cells. A172 cells were sequentially transfected with indicated siRNAs, and miRNA inhibitors. 24 hours later the cells were collected, and mRNA expression of the indicated cell cycle genes was determined by qRT-PCR.

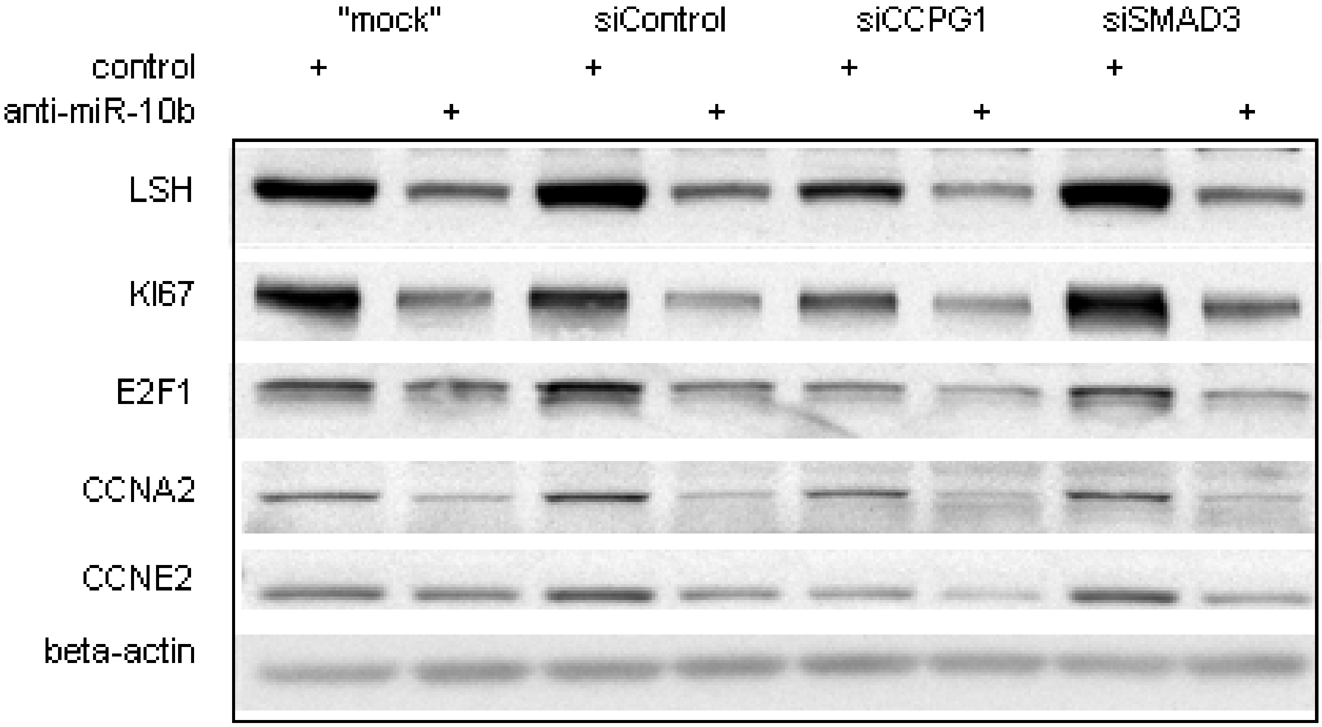

Supplementary Figure 8: The effects of CCPG1 and SMAD3 knock-down on cell cycle proteins in control and miR-10b depleted cells. A172 cells were sequentially transfected with siRNAs, and miR-10b inhibitor or non-specific oligonucleotide. Protein lysates were collected 24 hours later and analyzed for cell cycle proteins by Western blot.

Supplementary Table 1: Primers used in qRT-PCR reaction, 5′→3′

Pre-E2F1	F	TCACCTGGGCCTCAGTTTAC
	R	CTCAGGGCACAGGAAAACAT
E2F1	F	ACCCTGACCTGCTGCTCTT
	R	ATGGTCAGTTTCCAGGTCCA
p21 total	F	GACACCACTGGAGGGTGACT
	R	CTGCCTCCTCCCAACTCAT
pre-LSH	F	AGAGAGAGCGGAAGATGCTG
	R	GCAAAGAACACAAACCACACC
LSH	F	GACCCAGTCCGGAAGTGTAA
	R	TAAGCCATTCCATGCCTTCT
pre-CCNA2	F	CGAAGACGAGACGGGTAAAG
	R	TTCCAAAAGCCCAGGACAT
CCNA2	F	ACAAAGCTGGCCTGAATCAT
	R	ACTGTTGTGCATGCTGTGGT
pre-CCNE2	F	GTGCATGATTTCTTCCCATGT
	R	TGCTCTTCGGTGGTGTCATA
CCNE2	F	CCCCAAGAAGCCCAGATAAT
	R	AGGTGGCCAACAATTCCTAA
POLA1	F	TGAAGCTCACAGCGAACAGT
	R	TGCATCAAAATCTCCCTTCC
pre-POLE2	F	ACAACCAAAATCGGAGATGC
	R	CCGACATTTCATCAATCCAA
POLE2	F	CTGCTCCGTGGTGAAGCTAT
	R	GGGTTGCTTCTCAACTGCAT
pre-MKI67	F	CACCTGCTTGTTTGGAAGGT
	R	AATCCTAGAGCGCGTTTCTG
MKI67	F	GCTCGACCCTACAGAGTGCT
	R	CTCCTTCACTGGGGTCTTGA
PCNA	F	GCGTGAACCTCACCAGTATGT
	R	CCTGGTTTGGTGCTTCAAATA
TOP2A	F	GAATTGGAGGCTGTTGAAGC
	R	CGGAGAAGGCAAAACTTCAG
ORC1L	F	GATCTTCAGTGGTGCCATCC
	R	CGGATACGATGGGGAGTAGA
CDC6	F	CACTGGATGTTTGCAGGAGA
	R	CAACCCTCTTGGGAATCAGA

(*Continued*)

EZH2	F	TCATGCAACACCCAACACTT
	R	GAGAGCAGCAGCAAACTCCT
SMAD3	F	CAGTTGACCCGAATGTGCAC
	R	TCAGGTGCAGCTCAATCCAG
COL4A3BP	F	CAGGATGTAGGCGGAGATGC
	R	TTCCCAGTCATTGCGAACGT
PTPRR	F	GACAGACTTGCTTCCCAGGG
	R	CCATACGAAGCTGGCAGACA
FBXW7	F	GACCTCAGAGCAGCCAATGG
	R	GGTCCACTCCAGCTCTGAAA
CCPG1	F	TCAGCATGGGATTTGGCCAT
	R	TGTCCAACACCTTGCAAGATT
VTI1B	F	CGTTCTCATCGGATTGCCAC
	R	GCAGCTTGTTGGTTGTCACT
PLAUR	F	ACTCACGAACGCTCACTCTG
	R	GCTGAAACATCTGGGGCTCT
EPHA2	F	GAAGGTGGTGCAGATGACCA
	R	GGATCCCCACAGTGTTCACC
DLC1	F	CAAACTGCAACTGAGCCACC
	R	TTGTCGTCATCCTCTCAGCG
pri-miR-15a/16	F	TACGTGCTGCTAAGGCACTG
	R	CCATATTGTGCTGCCTCAAA
pri-miR-15b/16	F	GCGAATCATTATTTGCTGCTC
	R	CGTGCTGCTAGAGTGGAACA
pri-miR-17/18	F	CCTGCTGATGTTGAGTGCTT
	R	TGCCAGAAGGAGCACTTAGG
pri-miR-93	F	TACCTGCACGAACAGCACTT
	R	TGGGGTCTGTTTCACTCCAT
pre-GAPDH	F	TCACCAGGGCTGCTTTTAAC
	R	GGCTCACCATGTAGCACTCA
pre-ACTB	F	CGTCTTCCCCTCCATCGTG
	R	GGAAAGGACAAGAAGCCCTGA
ACTB	F	ATGATGATATCGCCGCGCTC
	R	TCGATGGGGTACTTCAGGGT
COX	F	CCCCTAATAATCGGTGCCCC
	R	CAGATGCGAGCAGGAGTAGG
GAPDH	F	ATGTTCGTCATGGGTGTGAA
	R	TGTGGTCATGAGTCCTTCCA

Supplementary Table 2: Primers for Chromatin Immunoprecipitation, 5′→3′

LSH	F	GCGAGATGACAGGATTTTCC
	R	CTCCTCTCAGAGCCTGCAAT
CCNA2	F	CCTGCTCAGTTTCCTTTGGT
	R	ATCCCGCGACTATTGAAATG
POLA1	F	TCCCTAAGGGGTCATCACAG
	R	AGCCTGAAAGCCAATCAGC
PCNA	F	GTCCGGAATATCCACCAATG
	R	TGGCTAATCGCACACTGAAA
MKI67	F	CGCTCCCTTCCTATTGGTC
	R	AGCCCTCCACTTCCTTCTTC
ORC1	F	TAGTTGGTGTCGCAACGAAG
	R	AGGACCAAAGCGTGTGTCTC
E2F1	F	CGCGTTAAAGCCAATAGGAA
	R	AAAGTCCCGGCCACTTTTAC
EZH2	F	GGGCCAAATAAAAGCGATG
	R	CTCCACTGCCTTCTGAGTCC
miR-15a/16 (DLEU2)	F	CATGCGTAAAAATGTCGGGAA
	R	GGTTATCCTGTCTCTCCCGCTAGATTGA
miR-15b/16 (SMC4)	F	GCAGGAGCGACAATAAGATGG
	R	CGCTCCTACCGGTGTTTCG
miR-17/92 (c13orf25)	F	CGCCCTTCATTCACCCACAT
	R	GAGAGCTTCGCGGAGGAG
miR-93 (MCM7) site1	F	CCGTCACTCATTCTAGGCCC
	R	GGGGAAGCTGAGAATCTCCG
miR-93 (MCM7) site 2	F	CGGAACAAAAGAACGCGTGT
	R	GCCTAGAATGAGTGACGGGG
APAF1	F	AGGGCAGCTTCTTCACCAG
	R	TCAGTGAAGCAACGAGGATG
CASP1	F	GCATATGCATGCACAGTGAGT
	R	GGCTTTTCTCTCCTCCCTTC
CASP7	F	GCCAGTTTGGCAGATTTGTT
	R	GCCCCTCGCTCTACAAAGTT
CASP8	F	CACAGTGCCAGGAAGTGAGA
	R	AACCTTTGCTCCAACCACTG
ACTB	F	AGCCTTGTACCGTGAACTGG
	R	CAGCGCGTAGTAGGTGTTCA

*miR-15a/16 (host gene DLEU2) and miR-15b/16 (host gene SMC4) E2F1 CHIP primer sequences were obtained from Ofir M, et al., 2011.
